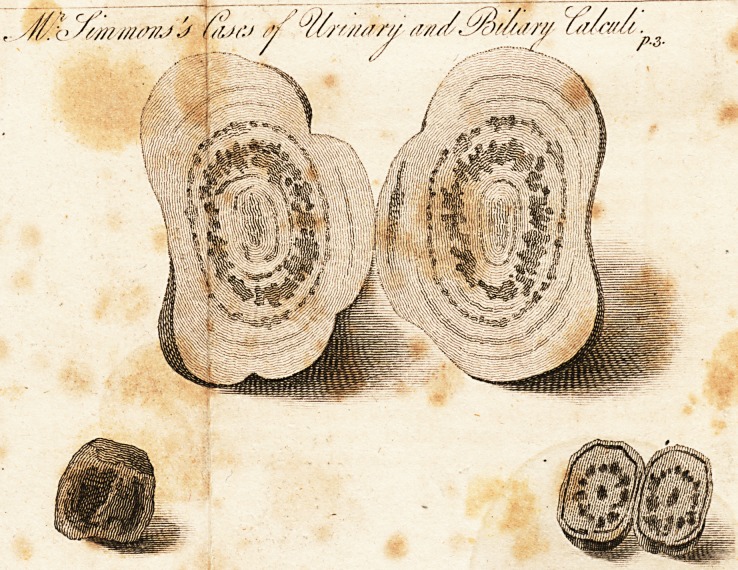# Observations on Select Subjects in Surgery

**Published:** 1806-07

**Authors:** 


### Medical and Phyſical Journal.

VOL. XVI.] JULY, 1806. [NO. 89.

*Printed for R. PHILLIPS, by W. Thorne, Red Lion Court, Fleet Street, London.*

### Observations on select Subjects in Surgery.

By Mr. Simmons.

( Continued from Vol. xv. pp. 1—8. )

### Sequel of the Case of Division of the Iris.

IN volume xv. page 4, I have narrated the circumstances of a case, in which I had used the couching needle of Mr. Hey with success, to divide a contraction of the iris. The enlargement of the pupil, it was said, had exposed a cataract, for the removal of which the necessity of another operation was apprehended. In this opinion, however, I was mistaken; for, a few days after the operation, on observing a ragged portion of the opake crystalline anterior to the iris, I advised delay, and finding at my subsequent examinations, that it still continued to diminish in a gradual manner, under an injunction to return to the Infirmary, I made him an out-patient. On the 24th of February, 1806, he came alone into the out-patients room, and told me that he could 'see.' The eye then presented no vestige of a cataract; the pupil was longitudinal in shape, transversely to the face; but the iris was still immovable.

Mr. Kite, in his paper on the 'Recommendation of Electricity, for the cure of cataract, illustrated by a case,' (London Medical Journal, Vol. vii. p. 146, Anno 1786) has the following passage: 'The real causes of these opacities are probably unknown; but when we recollect that the capsula of the crystalline humour is supplied with capillary vessels from some of the small ramifications of the ocular artery, and that these vessels have been seen running from the capsula into the body of the humour itself, we can easily conceive that inflammation and obstruction may, upon the application of certain exciting causes, just as readily occur in these vessels, as in those of the cornea, sclerotica, or any other part.'

Most commonly the cataract is slow in its formation ; at other times, however, it is formed with considerable rapidity, and, when confined to one eye only, it has happened that the loss of vision has been incidentally discovered by the patient himself.

Among the occasional causes of this malady, we may enumerate any sudden violence, such as a blow upon the eye; and this accident, which has proved the cause of this disease in one instance, has operated as a remedy in another, even where the opacity was apparently confirmed. In either of these cases, inflammation is the early consequence of the injury inflicted, but, in the one, an opake substance is deposited in the crystalline humour, which is thereby deprived of its transparency, and, in the other, the interstitial matter thus deposited, is again absorbed by the increased action of the vessels of the part which had been thus excited. This casual effort may therefore be intentionally imitated, by the topical application of electricity, æther, a solution of Cayenne pepper, or any other appropriate stimulant. I once succeeded in the dispersion of cataracts of four years duration, by dropping æther into the eyes, and drawing electric sparks from them, as strong as could well be borne by the patient. But it may be proper to mention, that the poor man who was the subject of these experiments, referred the origin of his complaint to the stroke of a flash of lightening.

A violent and long continued pain in the head, has frequently preceded the approach of cataract. And, although the pain has been felt in a distant part of the head itself, the seat of the disease has, I apprehend, been in the crystalline humour, similar to the pain which is felt at the point of the right shoulder, when the liver is materially diseased.

If we suppose a chronic inflammation to have existed in the crystalline humour, or in its capsule, we may explain the production of a cataract upon intelligible and well-known principles; and we can also understand how an acute inflammation will produce more quickly a similar result.

Upon this view of the subject too, a more just estimate may also be formed of the effects of the remedies to be employed, as the morbid consequences of an acute inflammation may be expected to be less obstinate to remove, than are those of the chronic kind. And it will likewise assist

Mr. Simmons's Cases of Intestinal Concretions. Vol. XVI. p. 3.

Mr. Simmons's Case of Nasal Polypus. Vol. XVI. p. 7.

Mr. Simmons's Cases of Urinary and Biliary Calculi. p. 3.

Printed for Richard Phillips New Bridge Street, Blackfriars, July 1. 1806.

assist us in explaining, how, by the breaking down of a cataract, by means of the couching needle, its dissolution may be expected, agreeably to the well-known principle of applying stimulants to, or even of making an incision down upon an indolent tumour, when external, in order to promote its absorption, and reduction.

### On certain Animal Concretions.

Calculi, or solid concretions, are not unfrequently met with in several of the cavities of the human body, such as the bladder of urine, the gall-bladder, and in the tube of the intestines. Examples of the two former are, however, most common; although instances of the latter are recorded by practical writers.

On a section of the urinary, and of the intestinal concretion, a nucleus to each, particularly the latter, is not uncommonly discovered; and hence we may refer the biliary concretion to a similar source. Any extraneous body which may have found its way into any of the above cavities, may become the nucleus of a stone; or even a particle of mucus, or of coagulable lymph, diverted from its original purpose in the system, may yield the rudiments on which calculous matter may concrete.

I do not recollect that a regular series of these several concretions has any where been published; in order therefore to supply this deficiency, I have procured a drawing to be made of a specimen of each of them.

### Urinary Concretion.

The urinary calculus, a section of which is here exhibited, was extracted from a man, about 60 years of age, in the year 1802; it weighed, within seven grains, two ounces avoirdupois; and was hard, and rugged on its outter surface. The radiated structure of the inner surface in one part, and the close compact texture in the other, will be seen in the drawing: but no vestige of a nucleus is discernible. It is probable therefore that the extraneous particle upon which the first crystals had formed, had consisted of mucus, or lymph, which had been comminuted and dissipated by the force of the subsequent crystallizations.

### Biliary Concretions.

It is not easy to conceive how an extraneous body should find its way into the gall-bladder, so as to become the nucleus of a biliary concretion; it is probable therefore, that a particle of inspissated bile constitutes the rudiment of this species of concretion.

The two specimens which are here exhibited, were taken out of the gall-bladder of a woman, who had died of the cholera morbus; there were four in number, nearly of an equal size, and all of them had a smooth polished surface, from attrition against each other.

One figure represents a biliary concretion entire; the other, the internal radiated structure of another concretion, exposed by a perpendicular section through the centre of it.

### Intestinal Concretions.

I have heard it recommended in conversation, to swallow the stones of certain kinds of fruit, in order to assist the digestion of the pulpy part of them. This vulgar error had not unlikely originated in the mechanical doctrine of digestion, now exploded; or a supposed analogy between the use of pebbles in the gizzards of fowls, and their action on grain, or other esculent substances. But we are now better informed by what process digestion is conducted; and are also assured, that such hard indigestible bodies, are so far from being useful, that they in many instances excite much disturbance in the first passages, before they are expelled; or lodge in the hollows of the intestines, until, by a gradual accretion, they are grown so large, as to prove the cause of ileus, or other fatal distemper.

The specimens here exhibited, were voided by a poor man, who, after a lingering and painful illness of several months duration, is since dead. The early symptoms of his complaint were a pain and circumscribed swelling on the right side of the body, about the head of the colon; together with symptoms of indigestion, and an irregular state of the bowels, which were sometimes loose, and at others constipated.

Soon after the expulsion of these concretions, the fullness in the side abated, leaving behind it a tensive cordy tumour, stretching to the extent of several inches, but which at length gradually diminished, and then totally disappeared. The hectic fever however, which had subsisted prior to their expulsion, was continued afterwards, and in the end sunk him.

What morbid changes had taken place in the abdominal viscera, I was prevented from ascertaining, the opening of the body having been refused.

On

### On a Case of Premature Delivery.

Practitioners have hitherto confined themselves to the exciting of premature delivery, to such cases of distortion of the pelvis as had previously been consigned to the use of the crotchet. This important fact was first promulgated by Mr. Barlow, of Bolton, (Med. Facts and Obs. vol. viii.) and it has since been confirmed by the experience of Mr. Hardman, of Manchester, (Medical and Physical Journal) and is now I believe pretty generally followed as a rule of practice in such cases. The preservation of the child is obviously the primary object for the bringing on of premature labour in the distorted pelvis; yet, if the safety of the mother, under particular circumstances, without distortion, should require similar means to be employed, with safety to the child, surely no good reason can be assigned why they ought not to be adopted. On this untrodden field I have lately ventured, and with a result entirely satisfactory.

Mrs. W——, a woman of a delicate habit of body, and the mother of many children, had, during all her former pregnancies, been much troubled with sickness and vomiting, and, contrary to what usually happens, both these symptoms had rather increased than diminished, after quickening, and continued until she was brought to bed.

Before this last pregnancy, she had not lain in of six years; yet even this long interval had produced no beneficial change in her constitution, for the former symptoms of sickness and vomiting recurred, and with increased violence. I was first requested to see her when she was about ten weeks off the full time; a cough, from which she was seldom free, was then much aggravated by a severe cold which she had caught; her stomach was in so irritable a state as to retain scarcely any thing taken into it, and she was tormented besides with an almost incessant cardialgia. For the cardialgia she had had frequent recourse to magnesia suspended in simple water, which gave some relief at the moment, and usually staid with her, but by the frequency of its repetition its laxative effect had proved considerable, and contributed very much to reduce her.

In the course of my attendance she took the carbonic acid gas, either in the effervescing draught, or as combined in Henry's soda water; lime and mint-waters, with and without colombo, absorbent and aromatic powders, the aqua kali, and other remedies; the stomach was embrocated with opiate and aromatic embrocations; and recourse was also had to blisters. Opium in a solid form was the only medicine from which she experienced any relief; and latterly the tincture of opium was administered by way of glyster. Nevertheless, the irritability of the stomach went on increasing, and even a single grain of solid opium was at length rejected, so that no food could be retained to supply the natural waste of the body.

In this state of the case, I apprized both her and her friends, that the only hope of her recovery rested on the bringing on of premature labour, the particulars of which I endeavoured, by explanation, to render intelligible to them. While they were coming to a determination upon the subject, nutritious and opiate enemas were injected, and baths of milk were applied to the lower extremities. However, they all soon became impatient, and even urgent for her to submit to my proposal; convinced that her existence must be short, unless she could thus obtain relief.

Upon this solicitation, six weeks from her full time, about eight o'clock on the evening of the 24th of March, 1806, I ruptured the membranes, an event which was denoted by the usual sign; at six in the evening following, she was taken in labour; and, at ten the same evening, she was delivered of a living child, after a labour, the other parts of the process of which were natural and easy.

From the time of her delivery, the sickness gradually abated, and she was enabled to retain some food upon her stomach; with the assistance of opium, her complaints by degrees left her; and, though still delicate, she is nearly restored to her former state of health. The child is still alive, and likely to live.

The principle of exciting premature delivery being once received, it may be easily transferred to other cases than those of distortion. In the present instance, the experiment has been fully justified, not only by the saving of the child, but by the preservation of the life of the mother, for which indeed it was here instituted. But notwithstanding my success in the present case, and my sense of duty to repeat the experiment under similar circumstances, I cannot too earnestly protest against the hasty adoption of such a measure. Language can ill convey a proper sense of my anxiety pending the decision; and I do entreat that it may be carried into effect only upon the most urgent necessity to preserve life, and after the most mature deliberation.

On

### On a Case of Polypus of the Nose.

The instances to be found in books of a large polypus extracted from the nose, are few. Indeed, for the most part, it is a complaint more troublesome than dangerous, and remediable by the most simple means. One species of polypus, however, is cancerous; and as cancer is still held to be the opprobrium of art, this species will accordingly stand as an exception to the general course of experience.

The specimen, of which an engraving of the natural size is here given, was extracted from the nose, or rather throat of a young man, about seventeen years of age; it had completely plugged up the posterior nostrils, and pharynx, and by pressing upon the epiglottis, had impeded respiration, as well as hindered deglutition.

In endeavouring to extract this polypus, the base of it being downwards, as represented in the drawing, I made long and strenuous efforts through the mouth, both with my fingers and with forceps, to detach it from its adhesions; and these efforts failing of success, I introduced a silver wire up the right nostril, and, through the mouth, drew it sufficiently low down to be passed over the base of the tumour; the wire was then alternately raised and tightened until I had carried it as near the root as I could. By means of the double tube, described in Mr. B. Bell's Surgery, I was then enabled to make a considerable degree of compression upon the part included within the ligature; this was still further tightened every day until on the fourth the polypus sloughed off without any hæmorrhage ensuing; and after exciting great fear of suffocation at the moment, it was ejected by an effort to vomit.

His breath was subsequently very fetid, and an ichorous discharge distilled down the throat, but he found a corrective for both in the inhalation of the carbonic acid gas.

Some time after the removal of the larger polypus, a smaller one, of a loose texture, appeared through the anterior nostril, and separated spontaneously.

As this case occurred fifteen years ago, and I have heard nothing of the patient since, I conclude, that the relief yielded by the operation was permanent.

## Figures and Tables

**Figure f1:**
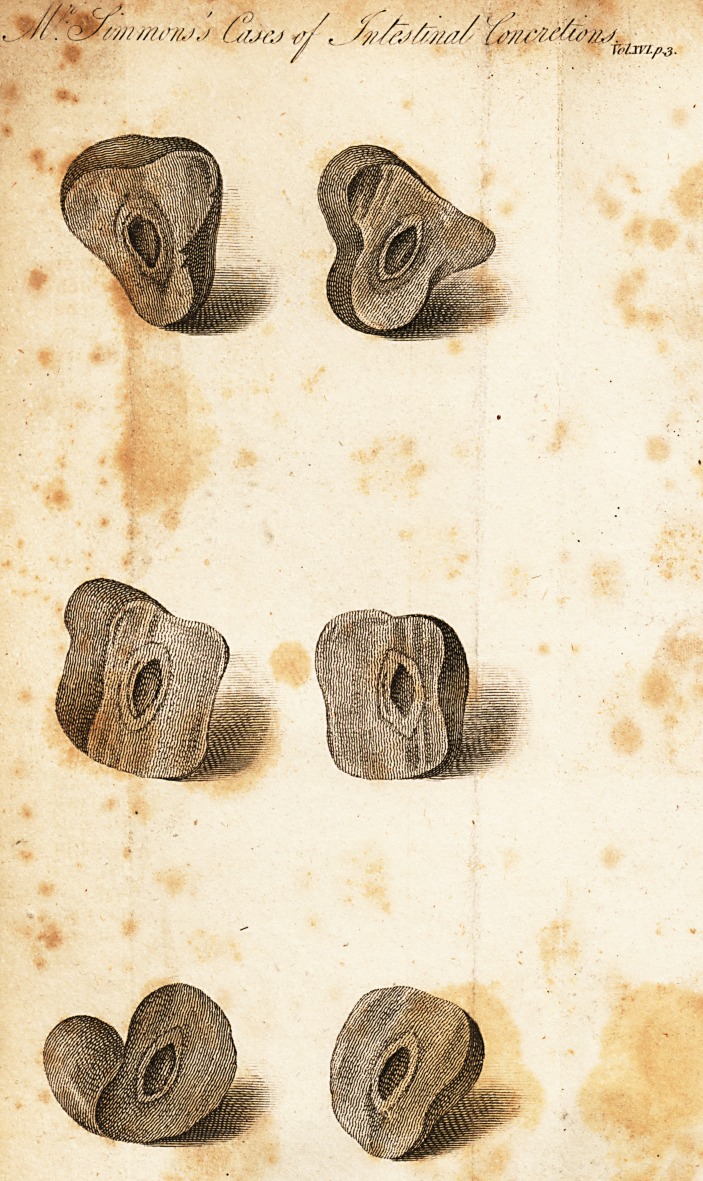


**Figure f2:**
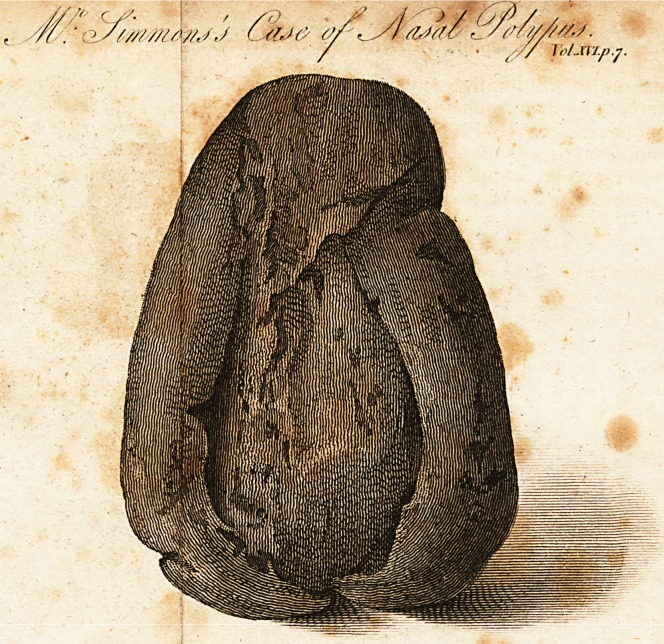


**Figure f3:**